# Construction of Data-Driven Performance Digital Twin for a Real-World Gas Turbine Anomaly Detection Considering Uncertainty

**DOI:** 10.3390/s23156660

**Published:** 2023-07-25

**Authors:** Yangfeifei Ma, Xinyun Zhu, Jilong Lu, Pan Yang, Jianzhong Sun

**Affiliations:** 1 College of Aerospace Engineering, Nanjing University of Aeronautics and Astronautics, Nanjing 211106, China; myff182011703@nuaa.edu.cn; 2 College of Civil Aviation, Nanjing University of Aeronautics and Astronautics, Nanjing 211106, China; zhuxinyun@nuaa.edu.cn (X.Z.); jilong_l@nuaa.edu.cn (J.L.); 3 College of Artificial Intelligence, Nanjing University of Aeronautics and Astronautics, Nanjing 211106, China; 0721_10320@nuaa.edu.cn

**Keywords:** performance digital twin, uncertainty quantification, anomaly detection, anomaly score

## Abstract

Anomaly detection and failure prediction of gas turbines is of great importance for ensuring reliable operation. This work presents a novel approach for anomaly detection based on a data-driven performance digital twin of gas turbine engines. The developed digital twin consists of two parts: uncertain performance digital twin (UPDT) and fault detection capability. UPDT is a probabilistic digital representation of the expected performance behavior of real-world gas turbine engines operating under various conditions. Fault detection capability is developed based on detecting UPDT outputs that have low probability under the training distribution. A novel anomaly measure based on the first Wasserstein distance is proposed to characterize the entire flight data, and a threshold can be applied to this measure to detect anomaly flights. The proposed UPDT with uncertainty quantification is trained with the sensor data from an individual physical reality and the outcome of the UPDT is intended to deliver the health assessment and fault detection results to support operation and maintenance decision-making. The proposed method is demonstrated on a real-world dataset from a typical type of commercial turbofan engine and the result shows that the F1 score reaches a maximum of 0.99 with a threshold of 0.45. The case study demonstrated that the proposed novel anomaly detection method can effectively identify the abnormal samples, and it is also possible to isolate anomalous behavior in a single performance signal, which is helpful for further fault diagnosis once an anomaly is detected.

## 1. Introduction

Critical engineering systems, such as aircraft gas turbines, must run safely and economically for their entire lifetimes. The anomaly detection and failure prediction of the gas turbines is of great importance for ensuring the reliable operation of safety-critical systems, which requires an accurate assessment of gas turbine conditions. Gas turbine condition assessment tracks measurable parameters during flight to derive insights into its current health state and trends for effective operation and maintenance decision-making [1].

Traditionally the condition of a gas turbine is assessed only based on discrete data points gathered during take-off and cruise, typically called engine performance snapshots [2]. Gas turbine performance trend changes usually trigger diagnostics alerts, which determine if an engine’s performance is changing from its normal operating range. These discrete data points can effectively reduce the amount of data required for analysis, however, provide very little information to reflect the variation in the performance state of the engine throughout the entire flight segment. The data sparsity related to snapshot data leads to difficulties distinguishing between faults and random scatters. Depending on the faulty component and the severity of the fault, it may need multiple data points to detect, which may cause false alarms and missed alarms. The resulting latency in fault detection based on performance snapshots may increase the risk of secondary damage [3,4].

In the field of gas turbine conditions assessment, the general fault detection schemes use thermodynamic engine models for computing reference values representing the nominal performance of the aircraft engine. Fault detection performs a comparison between these reference values and in-flight measurements. Significant deviations between the measurements and model predictions indicate an underlying fault. Such gas turbine performance models are traditionally derived from physical principles by domain experts [5]. The physics-based approach has the advantage of not requiring fault data to validate its performance, particularly in terms of explicability and ability to extrapolate. However, these models are costly to develop and usually are proprietary to the engine manufacturers, which are not available to the asset operators. An alternative paradigm is data-driven models developed directly from the operational data of an engine, where a flexible model structure is fitted to the system by training on historical data.

Currently, most airlines adopted Quick Access Recorders (QAR) for data acquisition, providing flight data continuously sampled at frequencies of 1 Hz and more, which is also referred to as full-flight data covering the whole flight. The availability of these data obtained from a large variety of sensors enables the introduction of new methodologies to assess engine condition, which offers the chance to detect engine faults within one flight more reliably to support more efficient in-service operations and maintenance decisions [6,7]. An approach for fault detection based on steady-state flight regimes of full-flight data is demonstrated in [8,9]. Weiss et al. proposed a steady-state fault detection framework with complete flight data using a one-class support vector machine, and high detection rates are demonstrated for various gas path component faults using synthesized datasets derived from full-flight data of commercially operated flights [6]. Hartwell et al. propose a practical and computationally inexpensive method for in-flight real-time anomaly detection based on a convolutional neural network. The efficacy of the method has been demonstrated on both real-time-series and synthetic snapshot data [7].

In recent years, the availability of big data from engineering systems has opened the era of digital twins, “defined as a virtual representation of a physical asset enabled through data and simulators for real-time prediction, monitoring, control and optimization of the asset for improved decision making through the life cycle of the asset and beyond” [10]. In the gas turbine field, it is a commonly used method to establish a digital twin for both the whole engine level and unit level based on the high-fidelity physical model to support more efficient in-service operation and maintenance decisions. Kraf et al. adopted a top-down method to construct a multilevel digital twin model from the component level to the whole engine, which can be used to support engine life consumption prediction to improve maintenance decisions [11]. Dawes et al. described a physics-based digital twin to support a through-life gas turbine service business model and they demonstrate how a digital geometry model can represent typical in-service component degradation and then support performance degradation prediction [12]. These papers demonstrate the application of digital twins from an MRO perspective and that the high-fidelity physical-based simulation is a necessary step to ensure a high-precision digital twin.

Another important application of the DT for gas turbines is to construct a performance digital twin for real-time control, performance monitoring, and fault detection. In the context of performance monitoring and anomaly detection, a well-known solution is to build an anomaly detector in which an underlying digital twin is constructed based on an adaptive physics-based thermodynamic model of the engine provided by manufacturers. Zaccaria et al. adopted an adaptive physics-based model as a performance digital twin for aircraft engine fault detection and isolation [13]. Panov et al. proposed a gas turbine PDT for real-time control and monitoring functionalities based on a physics-based performance model and real-time embedded computing [14]. In general, these physics-based models are proprietary to manufacturers and usually unavailable to the asset operators, an alternative is to construct data-driven performance DT models on operating data from an in-service physical asset. However, constructing such a performance DT covering various stationary and non-stationary operating conditions requires massive amounts of historical flight data and complex deep models.

As an important enabling technology of data-driven Digital Twin, machine learning, especially deep learning, has recently gained attention due to its ability to learn fault patterns directly from raw sensor data and its capacity to handle non-linearity in complex temporal correlation [15,16]. In the context of anomaly detection, an anomaly detector is typically built on an underlying data-driven DT trained on real-time operating data from a physical asset to judge when it deviates from its normal behavior [15]. Xu et al.. proposed a digital twin-based anomaly detection approach (ATTAIN: Anomaly deTection with digiTAl twIN)) which continuously and automatically builds a digital twin with live data obtained from a Cyber-Physical System that implements a Generative Adversarial Network to detect anomalies [15]. Castellani et al. introduced a Digital Twin-based anomaly detection method that is tailored for weakly supervised settings with very few labeled data samples. The method is demonstrated on real-world use-case data, and the developed solutions outperform state-of-the-art anomaly detection approaches [17]. It has shown high-grade performance because of its power to deal with unstructured and unlabeled data, which is of great significance for constructing aero-engine condition monitoring digital twins.

Currently, most airlines adopted Quick Access Recorders (QAR) for data acquisition, and the availability of these big data sets enables the introduction of new PDT-based approaches in engine condition monitoring. Data-driven Performance Digital Twin (PDT) is an important method to accomplish real-time condition monitoring covering the full flight of an engine under various operating and environmental conditions. However, the research on the PDT modeling of the aircraft engine covering the whole flight conditions is very limited. This study aims to develop a novel approach to anomaly detection based on the digital twin paradigm that is an accurate simulation of an individual ’as-operated’ gas turbine. The developed digital twin consists of two parts: the Digital Twin model and fault detection capability. The Digital Twin model is a digital representation of the expected behavior of a real-world gas turbine, named Performance Digital Twin (PDT), which capitalizes on multivariate time series data obtained from the physical asset in operation to enable gas turbine performance tracking. Depending on the context, a digital twin can provide various capabilities; in this article, we focus on the anomaly detection capability of a digital twin. The main contributions of this paper is to propose a novel data-driven performance Digital Twin with uncertainty quantification, denoted as uncertain performance digital twin (UPDT), just based on the real-world gas turbine operational data rather than the physics-based model. The proposed UPDT produces a probabilistic digital representation of the expected performance behavior of a real-world gas turbine. Then based on the UPDT, the fault detection capability is developed and a novel anomaly measure based on the first Wasserstein distance is proposed to characterize the full flight data to detect anomaly.

The remainder of the article is organized as follows. Section 2 presents an overview of the proposed framework and Section 3 describes the LSTM-AE-based scheme for performance digital twin. The PDT uncertainty quantification and fault detection measures are discussed in Section 4 and Section 5. A case study is carried out to demonstrate the developed method in Section 6. Finally, a summary of the work and outlook are given in Section 7.

## 2. Fault Detection Framework Based on UPDT

**Figure 1:**
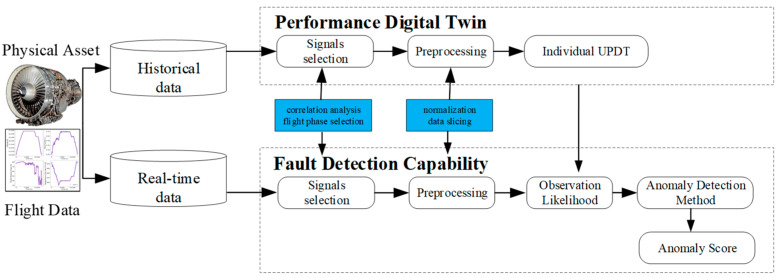
Proposed Fault Detection Framework Based on UPDT.

The developed fault detection framework based on the performance digital twin with uncertainty quantification is presented in Figure 1. The developed framework comprises two parts: Uncertain performance digital twin (UPDT) and fault detection capability. UPDT aims to produce a probabilistic digital representation of the expected performance behavior of a real-world gas turbine operating under various conditions during a flight directly from raw sensor data. The replica can be used to simulate and predict the engine’s behavior at different ambient/operating conditions. Although simulation of the whole gas turbine is feasible, interesting subset signals, such as the operating conditions and gas path key performance parameters, are selected to form a performance digital twin based on engineering knowledge. The UPDT is first trained based on nominal historical data and then continuously learns from new data to improve its anomaly detection performance. The UPDT, a probabilistic simulation of an individual ’as-operated’ gas turbine, is then used to predict the performance parameters with confidence intervals for quantifying uncertainty in the models.

Fault detection capability is developed based on detecting UPDT outputs that have low probability under the training distribution, i.e., the Out-of-Distribution (OOD) detection mechanisms which have received remarkable attention in recent years [18]. An anomaly detection model, such as a density model of normal representations [19], a model of distances from some nominal samples [20], or a model of reconstruction errors [21], is created to compute an anomaly score. A threshold can be applied to this score in order to discriminate samples into fault and health. Density-based methods attempt to model the distribution of normal data with the assumption that the anomaly sample has a low likelihood. In contrast, the normal sample has a higher likelihood under the estimated density model. In this study, a density-based method is used to explicitly model the nominal historical data covering expected operating conditions with a multivariate Gaussian distribution and flag test data in low-density regions as anomaly samples based on their likelihoods.

## 3. Gas Turbine Performance Digital Twin

Traditionally, the gas turbine performance digital twins are based on an adaptive physics-based performance model provided by the engine manufacturers. Since these models are proprietary and usually unavailable to the asset operators, to circumvent the practical constraints of implementing physics-based PDT for airline companies, an alternative is given by data-driven models to develop data-driven PDT directly from the operational data of an engine. One of the key characteristics of a Digital Twin is the interconnection of information between the digital entity and the physical reality. In this paper, this is achieved through the use of sensors of the gas turbine which can directly measure the engine operating, control and performance parameters. The information exchange from the virtual representation to the physical reality is through informed decision-making, such as engine performance degradation assessment and fault detection to support operation and maintenance planning.

Constructing such a PDT covering various stationary and non-stationary operating conditions requires correctly assessing the temporal correlations in full-flight data. Currently, most airlines adopted Quick Access Recorders (QAR) for data acquisition, providing flight data continuously sampled at frequencies of 1 Hz and more during the whole flight, which is also referred to as full-flight data. The availability of these data sets enables the introduction of data-driven PDT–based approaches in engine condition monitoring.

The PDT aims to produce a digital representation of the expected performance behavior of a real-world gas turbine operating under various conditions during a flight. To correctly assess the transient performance of gas turbine engines requires the previous data points to be considered resulting in an auto-correlation. It is necessary to build models to capture time sequence information in the data. In the following, LSTM based deep learning network [22,23], which is well-suited for modeling sequential data with the temporal correlations in full-flight data, is implemented to model the steady-state and transient performance of gas turbines. LSTM is well-suited for sequence learning tasks and has been implemented for encoder and decoder networks for anomaly detection due to the capability of LSTM to model sequential data with temporal information [24].

Autoencoder is an unsupervised learning algorithm that attempts to replicate its input to its output. The hidden layer *h* inside the algorithm can describe a certain code for the input’s representation. It consists of an encoder and a decoder and is mainly applied for dimensionless feature extraction. The main function of the encoder is shown below:

h=f(W⋅x+b)

where *f* contains a linear change *W* and a nonlinear activation *b*. The decoder converts the hidden representation h to the initial input in a similar manner, as shown below:

$$x^{*}=g(W^{\prime }\cdot h+b^{\prime })$$

Here the parameters $\delta =[W,b,W^{\prime },b^{\prime }]$ can minimize the cost function. For AE, the form of intermediate layers is noteworthy. The algorithm should be able to predict the target signal y, so the encoding *h* should carry the interrelationship of different sensor parameters. The aim of the learning process is to minimize a loss function *L* as far as possible. The specific loss function for the proposed LSTM-AE based scheme will be discussed in Section 4.

**Figure 2:**
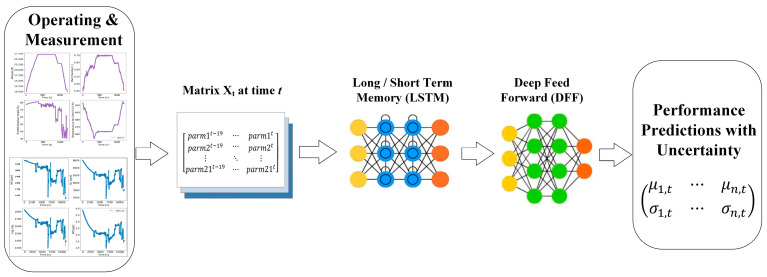
LSTM-AE based scheme for uncertain performance digital twin.

The combined framework of LSTM-AE is suitable for constructing the data-driven PDT due to its advantage in processing time-series data. The scheme of the proposed LSTM-AE based PDT is shown in Figure 2. The architecture allows the streaming of data from selected sensors of a gas turbine in real-time into the developed PDT. To account for the temporal correlation, a temporal feature extraction utilizing LSTM neural network is used as a preprocessing step. A fixed-size sliding window technique is applied to create the input samples of the PDT. Input data is treated as a 2D window with length *T* and width *S*, where *T* and *S* are time steps and the number of selected signals. Each input sample is recorded as *X_t_*, and *X_t_* is a matrix of *j* × 1, while each column *j* represents a signal at time *t*. The matrix *X_t_* here can be regarded as a “window” that moves on data series, and the elements inside the window represent the condition of the aero-engine within a specific time interval. This technique allows the trend information to be preserved, which is suitable for processing dynamic and variable time series data.

## 4. PDT Uncertainty Quantification

The digital twin incorporates as-operated data of the physical product to assist in the predictive and decision-making process. The goal of the PDT training is to learn accurate reconstruction of the normal performance behavior of the engine under continuously varying flight conditions. However, the lack of knowledge about the uncertainty of data captured from the physical domain, and consequently of models created from them, has a great impact on how much a PDT conforms to its physical product. In general, uncertainty is classified as epistemic or aleatoric. Epistemic uncertainty relates to the lack of knowledge, caused by poor assumptions, poor models and missing data. On the other hand, the aleatoric uncertainty relates to the variability of physical processes, which is inherent to the non-deterministic nature of measurement processes [25]. Calculation of the uncertainty is complex due to the large number of factors affecting it.

To improve the reliability and robustness of fault detection, the data-driven PDT should produce a probabilistic digital representation of the expected performance behavior of a real-world gas turbine. In this paper, a novel Uncertain Performance Digital Twin (UPDT)is proposed to take into account various uncertainty, such as operating condition disturbances, engine dynamics as well as measurement uncertainty. In the following, the performance prediction uncertainty quantification is taken into account in the deep neural networks to achieve a more reliable and robust fault detection.

For modeling the uncertainty, the gas turbine performance measurements are assumed to be sampled from a given probability density function $P\left(Y_{t}|\theta \right)$. The parameter θ of the probability density function characterizing the performance prediction is then estimated by the UPDT based on input *X_t_*. The estimation of the distribution of the output data $Y_{t}$ is central to the following anomaly detection scheme. In this work, output distribution is described using a multivariate Gaussian with mean $\mu_{t}$ and correlation matrix $\sum_{t}$ given by

$$p\left(\vec{y}|\vec{\mu },\sum \right)=\frac{1}{\left|\sum \right|\sqrt{{\left(2\pi \right)}^{n}}}exp(-\frac{1}{2}{\left(\vec{y}-\vec{\mu }\right)}^{T}\sum \left(\vec{y}-\vec{\mu }\right))$$

$$\vec{\mu }={\left[\mu_{1},\cdots ,\mu_{i},\cdots ,\mu_{n}\right]}^{T}$$

$$\sum =\left[\begin{array}{ccc} \sum_{1,1} & \cdots & \sum_{1,n}\\ \vdots & \ddots & \vdots \\ \sum_{n,1} & \cdots & \sum_{n,n} \end{array}\right]$$

To reduce the complexity of the artificial neural network and, therefore the total number of parameters to be estimated, the performance measurements are considered to be sampled independently, leading to uncorrelated measurement noise and, therefore, negligible cross-correlations $\sum_{i,j}$. This simplification collapses the correlation matrix ∑  into a diagonal matrix ∑ = *diag* ($\sum_{1,1}$, …, $\sum_{n,n}$).

The UPDT model is trained through a mini-batch stochastic gradient descent approach to reconstruct the value of the target parameter *Y_t_* at the current moment with uncertainty quantification. Optimization of the weights of a deep neural network requires an optimization target. The objective function is defined as maximizing the likelihood of observing the data *Y_t_* underlying the chosen probability density function $P\left(Y_{t}|\theta \right)$, which is equivalent to the minimization of the negative log-likelihood:

$$NLL=-log\left(P\left(Y_{t}|\theta \right)\right)$$

The goal of the proposed UPDT in this work is to accurate reconstruction of the normal behavior of the engine with uncertainty estimation under continuously varying flight conditions. Various approaches to uncertainty estimation in deep neural networks are available, such as using dropout at run-time [19], using ensembles as a prediction scatter and Bayesian neural network solutions [26]. In this work, the explicit estimation approach is used, which allows us to use a specific probability density function to characterize the estimation uncertainty while retaining the flexibility of non-Bayesian neural networks.

## 5. Fault Detection Capability

Fault detection capability is developed based on detecting UPDT outputs that have low probability under the training distribution. A density-based anomaly detection model is created to compute an anomaly score. For some instances x, these methods then yield an outlier score $D_{f}\left(X\right)$. A threshold τ can be applied to this score in order to discriminate samples into anomaly and health [18].

(1) $$\mathrm{Anomaly}(X)=\left\{\begin{array}{c} 1\;if\;D_{f}\left(X\right)>\tau \\ 0\;else \end{array}\right.$$

Density-based methods attempt to model the distribution of normal data, with an assumption that the anomaly sample has a low likelihood whereas the normal sample has a higher likelihood under the estimated density model. In this study, a density-based method is used to explicitly model the historical nominal data covering expected operating conditions with a multivariate Gaussian distribution, and flag test data in low-density regions as anomaly samples based on their likelihoods.

Data scatter will always be present in the full flight data because of measurement system accuracy, recording accuracy, and actual stability of the aircraft engine during the data acquisition. Since there will always be a certain number of statistical outliers during one flight, an anomaly score, $D_{f}\left(V\right),$ characterizing the full flight data is proposed based on the first Wasserstein distance [27]:

(2) $$D_{f}\left(V\right)=log(l_{1}\left(U,V\right))=log(\underset{\pi \in \Gamma (U,V)}{\inf}\int_{R\times R}^{}\left|x-y\right|d\pi \left(x,y\right))$$

where Γ(U,V) is the set of (probability) distributions on R×R whose marginals are U and V on the first and second factors respectively. The first Wasserstein distance, also known as the earth mover’s distance, computed distance between two 1D distributions, where the input distributions can be empirical, therefore coming from samples whose values are effectively inputs of the function. In this study, *U* comes from the training samples of the historical nominal data, and *V* comes from the test sample of one flight. If the outlier score exceeds a predefined threshold τ, the outliers are no longer considered statistical but systematic, indicating a fault.

## 6. Case Study on Real-World Gas Turbine

### 6.1. Data Sets

The case study is based on a real word dataset from a typical type of commercial turbofan engine, which is collected from a twin-engine aircraft fleet operating mainly for domestic routes over about two years. The engines are operating under various conditions, each of which has served around 3000 flights during this period. The average duration of each flight is about 2 h. Full-flight operation data consisting of continuous and discrete parameters defining the environmental conditions, power settings, and controller settings are acquired and stored in QAR. This data set contains engine dynamics and real word disturbances under actual operating conditions. For the engine type studied in this paper, the example operating and performance parameters related to engine operation recorded in the QAR are listed in Table 1.

**Figure 3:**
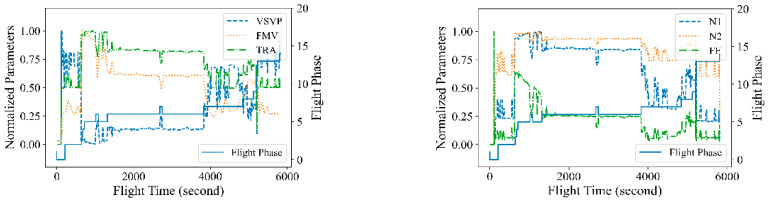
The time series data from heterogeneous sensors of turbofan during one flight.

The example parameters of the entire flight are shown in Figure 3. Each of the parameters is on different scales and has different acquisition frequencies. Due to the different acquisition frequencies of the parameters, it caused some null values in the dataset. Here linear interpolation is used to fill these null values. The influence of dimensionality among the different parameters should be eliminated to improve the performance of the deep learning network. A data normalization step is carried out to homogenize the variables into a common scale using the equation as follows:

(3) $$x_{i}^{\prime }=\frac{x_{i}-\mu_{i}}{s_{i}}$$

where $\mu_{i}$ is the mean value of the *i*-th sensor parameter, and *S_i_* is the standard deviation.

### 6.2. UPDT Construction for a Turbofan Engine

The real QAR data of the turbofan engine is used to train and test the UPDT model. For modeling and training, seven parameters (SAT, M, ALT, N1, TRA, VSVP, VBVP) characterizing the engine operating conditions and thrust setting are selected as the input data, and three parameters (EGT, FF, N2) characterizing the engine performance are used as the target output. A fixed-size sliding window technique is applied to create the input samples for the UPDT to account for the temporal correlation of the data.

The network layer structure of the proposed data-driven UPDT in this article is shown in Table 2. The UPDT model is compiled and configured in Keras by Python, and the hardware condition is a Dell workstation with specification parameters as Intel (R) Xeon (R) Gold 5122 CPU @ 3.60 GHz, 128 GB of RAM, NVIDIA GeForce RTX 2080 Ti GPU, and 64-bit operating system of the windows-10. Adam is selected as the optimizer, and the negative log-likelihood is selected as the loss function.

The training data set is constructed based on 300 flights randomly selected from around 3000 flights covering two years of operation of one turbofan engine, with a total of 1,180,000+ training samples. Then the data from 29 flights randomly selected from the remaining data are used for the test, including 26 normal flights and three known abnormal flights. For a comprehensive investigation of the proposed fault detection framework underlying various fault modes, 120 synthetic datasets were generated by the superimposition of the three key performance measurements with theoretical parameter shifts generated using the engine technique manual provided by the manufacturer. The dataset used for model training and test are listed in Table 3. Table 4 presents the theoretical parameter shifts for 12 typical fault modes around a specified cruise operating point from the engine manufacturer technique manual.

### 6.3. Results and Discussion

**Figure 4:**
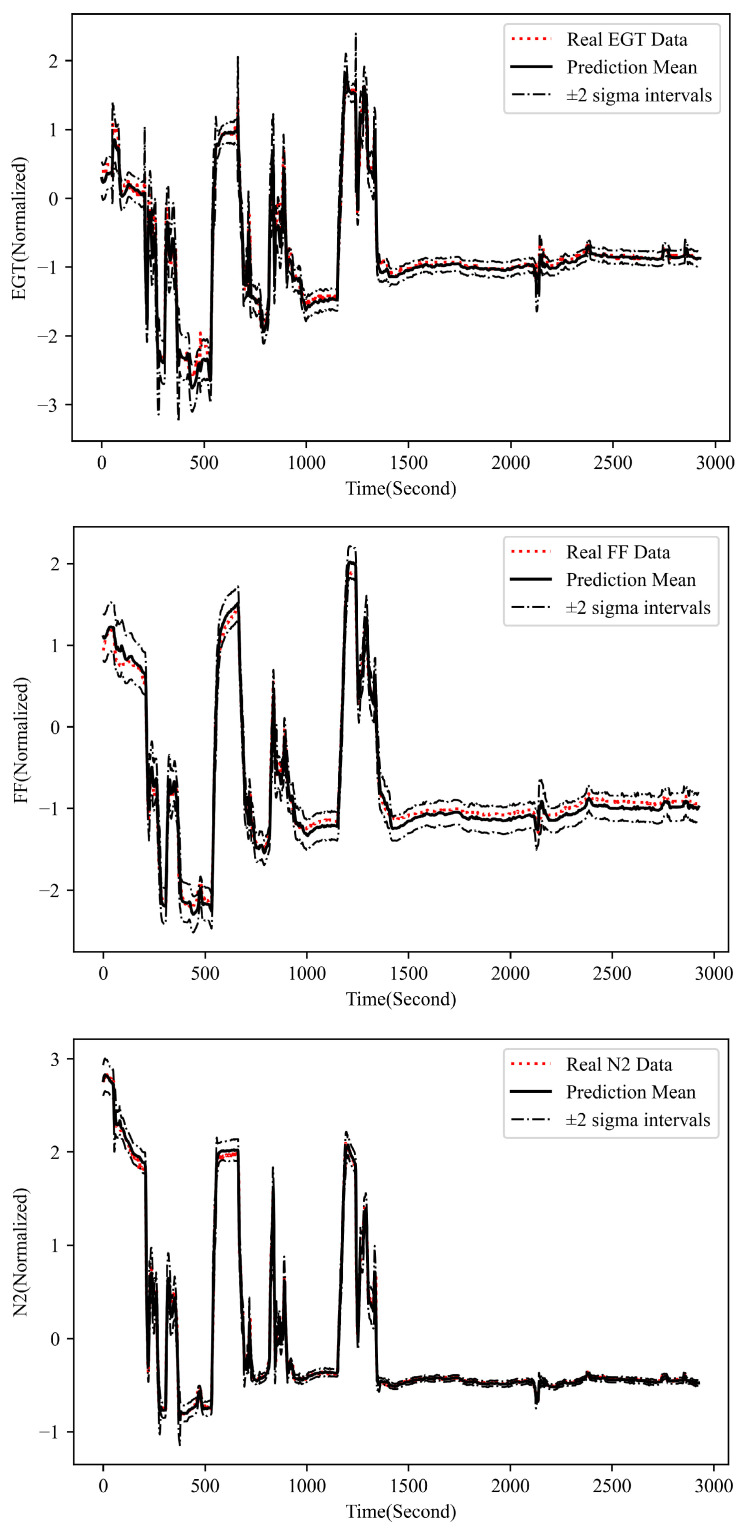
Key performance parameters reconstruction with uncertainty estimation during one normal (Flight No. 8).

With the well-trained UPDT, we can get the reconstructed key performance parameters with uncertainty estimation ($\mu_{t}$, $\sum_{t}$) and the observation likelihoods $P\left(Y_{t}|\left(\mu_{t},\sum_{t}\right)\right)\;$ based on the real measurements $Y_{t}$. Then, the outlier score $D_{f}\left(X\right)$ characterizing the entire flight data is calculated using Equation (3). Figure 4 presents the reconstructed three key performance parameters, i.e., EGT, FF and N2, with uncertainty estimation during one flight based on the developed UPDT. It can be seen from the figure that the proposed data-driven UPDT can well characterize both the steady and transient performance behavior of the turbofan engine under various operating conditions. It means the developed UPDT trained based on a dataset from 300 normal flights can produce a probabilistic digital representation of the expected performance behavior of a real-world gas turbine operating under various conditions during a flight. The performance prediction uncertainty quantification is also taken into account in the UPDT to achieve a more reliable and robust fault detection.

**Figure 5:**
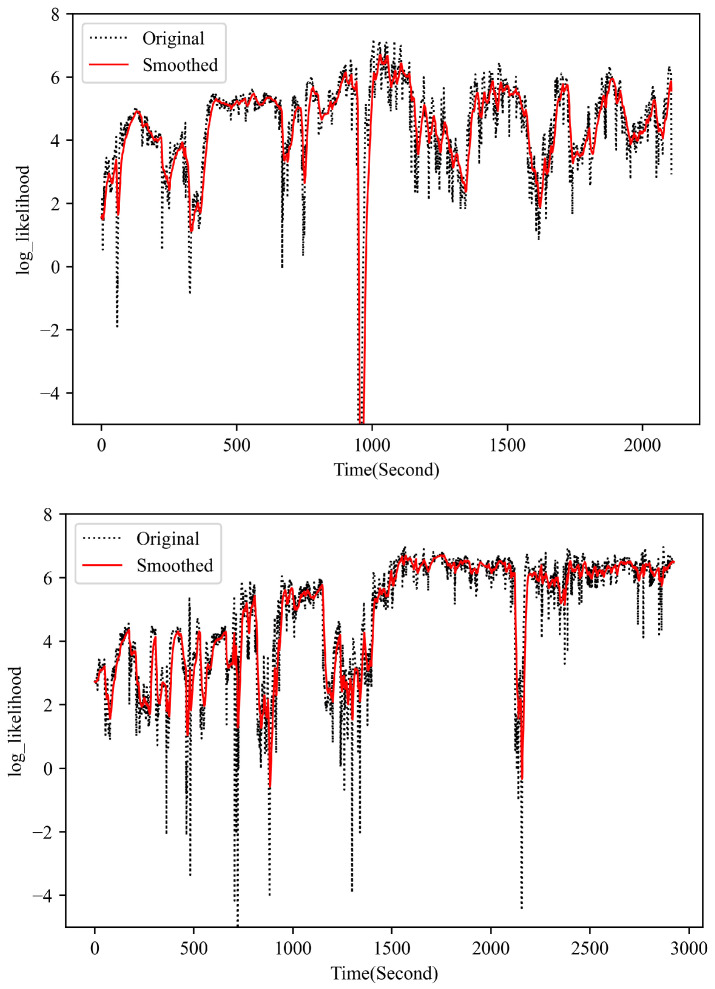
The observation log likelihood for multivariate measurement during two normal flight (Flight No. 8 and 17).

**Figure 6:**
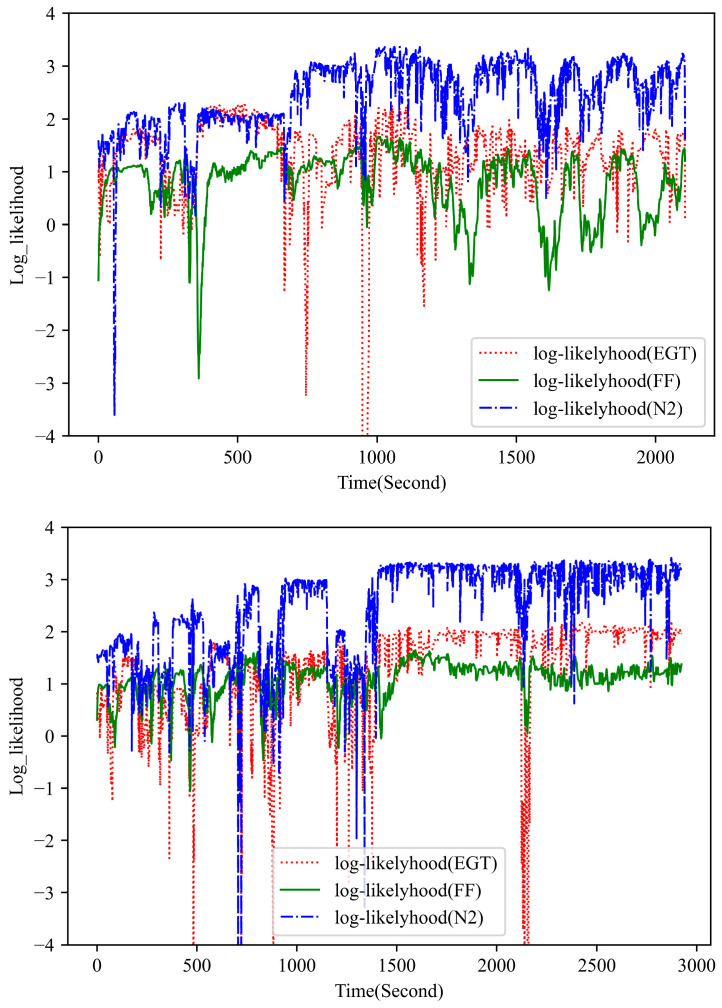
The observation log likelihood for single parameter during two normal flights (Flight No. 8 and 17).

To further demonstrate the parameter reconstruction performance of the developed UPDT, the observation log-likelihood of the multivariate parameter (i.e., $P\left(Y_{t}|\theta \right)$) based on the real measurement during the flight is presented in Figure 5. As shown in Figure 5, during the most time of the flight, the observation log-likelihood of the multivariate measurement is positive, which indicates a normal performance behavior of the engine as expected. As explained above, the developed UPDT is trained to attempt to model the distribution of normal data of the engine under various operating conditions, with an assumption that the anomaly sample has a low likelihood whereas the normal sample has a higher likelihood under the estimated density model. Some spikes and low likelihood regions in the plot can be mainly attributed to the measurement system accuracy, model accuracy, and stability of the aircraft engine during the data acquisition. It demonstrates that the developed UPDT performs well to reconstruct the performance measurements of normal flight with uncertainty quantification. To further check the engine performance behavior, the observation likelihood for each parameter measurement is calculated shown in Figure 6. As shown in the plots, each signal has a different observation likelihood of its measurement during the flight, which indicates that it is possible for the proposed method to isolate anomalous behavior in a single signal, which is helpful for the subsequent fault diagnosis once an anomaly is detected.

**Figure 7:**
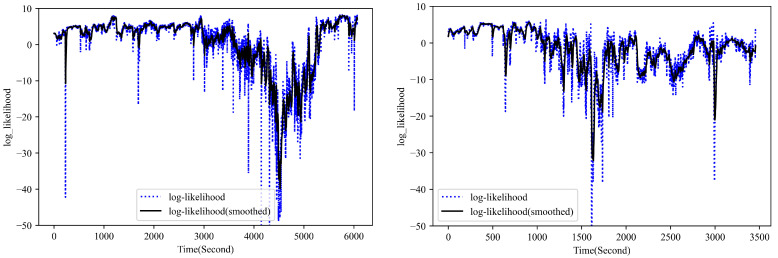
The observation log likelihood for multivariate performance measurements during two anomalous flights (Left plot for a known fault and Right plot for a simulated fault).

**Figure 8:**
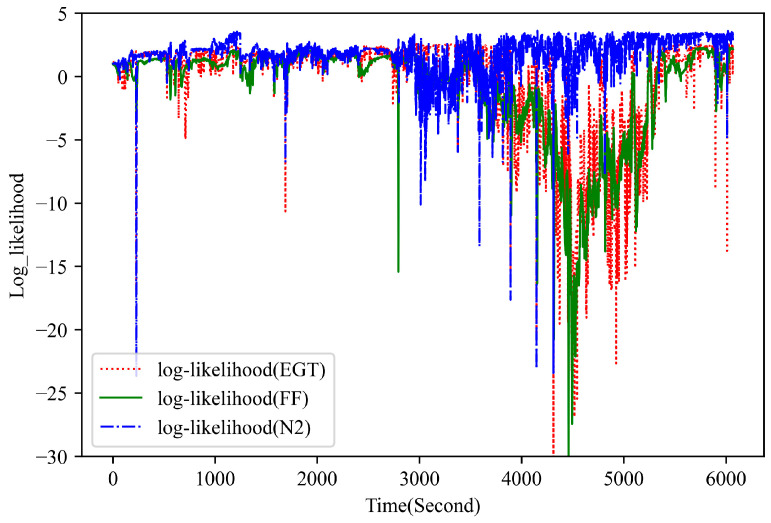
The observation log likelihood for single signal during one known anomalous flight.

Figure 7 presents the observation log-likelihood for multivariate measurements during two abnormal flights. In this study, the UPDT is trained to explicitly model the distribution of the normal historical data, with an assumption that the anomaly sample has a low observation likelihood, whereas the normal sample has a higher likelihood. Compared with the normal plots in Figure 5, the proposed method clearly detects the anomalous behavior of the flight data with a relatively lower observation likelihood. Figure 8 demonstrates the observation log-likelihood for each signal during one known anomalous flight. It can be observed that the flight anomaly is mainly attributed to the unusual behavior in the EGT and FF signal, which is helpful for the subsequent fault diagnosis.

**Figure 9:**
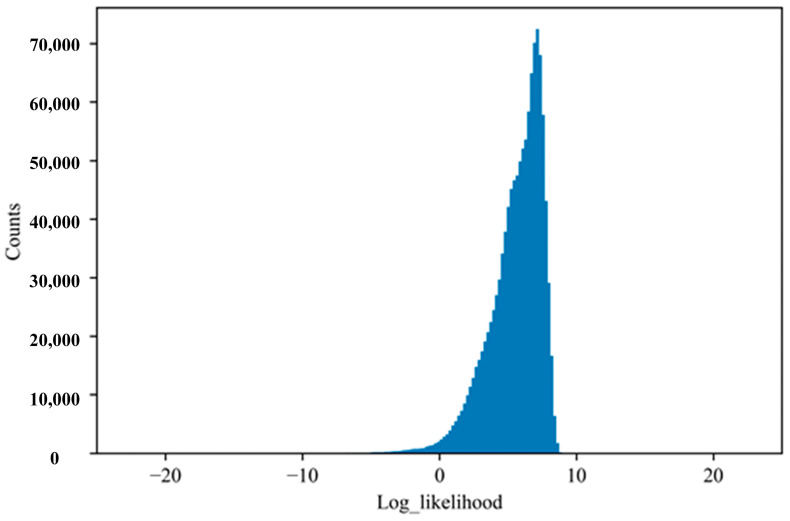
The histogram of log likelihood for training dataset (U).

**Figure 10:**
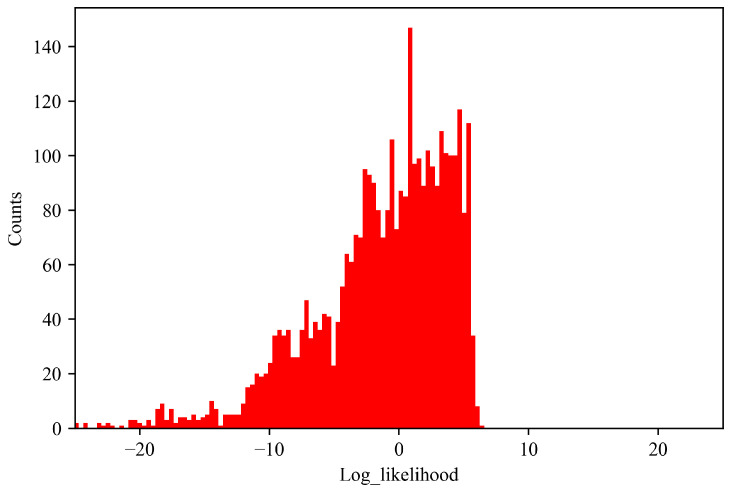
The histogram of log likelihood for one known anomalous flight (V).

To further demonstrate the proposed observation likelihood, the histogram of the observation log-likelihood for the training dataset (U) from the historical normal flights is presented in Figure 9. It can be observed most of the log-likelihoods for the multivariate measurements are positive, which indicates a normal performance behavior of the engine. For comparison, Figure 10 presents the histogram of the log-likelihoods of the multivariate measurements from one known anomalous flight (V). Obviously, the log observation likelihood for the flight containing the known fault is observed to be distributed differently from the historical normal flights. It can be observed the known fault flight (shown in Figure 10) has a large amount of mass at a much lower log-likelihood than the nominal flights (shown in Figure 9), which is no longer considered statistical but systematic, indicating a fault. This can be explained that the anomaly sample has a low likelihood whereas the normal sample has a higher likelihood under the developed UPDT model.

**Figure 11:**
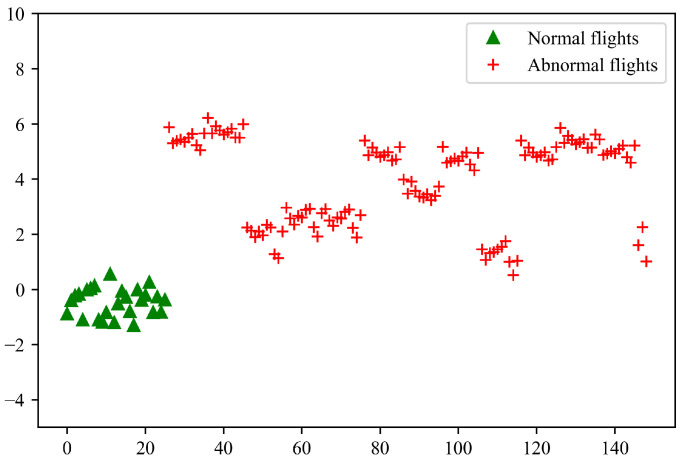
The anomaly scores for the test flights.

**Figure 12:**
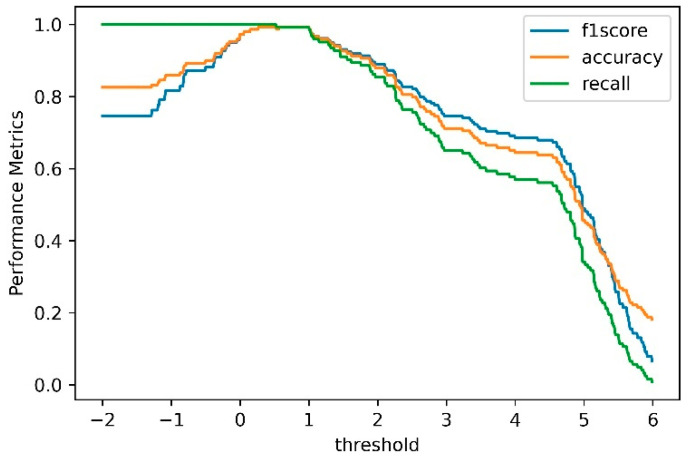
The performance metrics with different threshold.

The proposed anomaly score derived from the first Wasserstein distance is then calculated to characterize the full flight data. The anomaly score indicates the difference between two 1D distributions of the log-likelihood computed based on the training and test dataset, respectively. If the anomaly score exceeds a predefined threshold, i.e., τ, the outliers are considered a fault. Figure 11 shows the computed anomaly scores for the test flights, i.e., the log Wasserstein distance. It is clear that the proposed anomaly measure can effectively separate the normal and abnormal samples, and as expected the anomalous flights have greater distance than the historical normal flights. Figure 12 shows the accuracy, recall and F1-score with different τ values for the proposed anomaly detection method. In the case of a small τ, all the test samples are marked as anomalies. As the τ value increases, the F1 score increases and reaches a maximum at τ=0.45 with an F1 score of 0.99.

## 7. Conclusions

This work presents a novel anomaly detection approach for gas turbine engines based on a data-driven performance digital twin. The digital twin consists of an uncertain performance digital twin (UPDT) and fault detection capability. UPDT is a probabilistic digital representation of the expected performance behavior of real-world gas turbines operating under various conditions. Fault detection capability is developed based on detecting UPDT outputs that have low probability under the training distribution and flag test data in low-density regions as outliers based on their observation likelihoods.

The proposed method is demonstrated on a real-world dataset from a typical type of commercial turbofan engine. The case study result shows the proposed data-driven UPDT can well characterize both the steady and transient performance behaviors of the real-world turbofan engines under various operating conditions. A novel anomaly measure based on the first Wasserstein distance is proposed to characterize the full flight data, and a threshold to this measure is determined in order to detect anomaly flight. The case study result shows that the F1 score reaches a maximum of 0.99 with a threshold of 0.45. One of the limitations of this work is that only three key parameters (EGT, FF, N2) are used to characterize the engine performance. Therefore, a potential future research direction is developing a UPDT that can take more performance parameters into account and the cross-correlations among the parameters need to be further explored.

## Figures and Tables

**Table 1:** Example engine parameters recorded in QAR.

Parameter	Unit	Parameter	Unit
Static Air Temperature (SAT)	°C	Exhaust Gas Temperature (EGT)	°C
Altitude (ALT)	Feet	Thrust Lever Angle Resolver (TRA)	degree
Mach number (M)	Ma	Selected FMV Position (FMV)	-
Low-pressure rotor speed (N1)	%	Fuel Flow (FF)	kg/h
High-pressure rotor speed (N2)	%	Fuel Air Ratio (FARS)	-
HPC Exit static Press (PS3)	kPa	Selected Oil Pressure (OILP)	-
HPC Inlet Temperature (T25)	°C	Selected VSV Position (VSVP)	degree
HPC Exit Temperature (T3)	°C	Selected VBV Position (VBVP)	-
Engine Bleed	-	Engine Cowl Anti-ice switch	-
T/R Deployed	-	Wing Anti-ice switch	-

**Table 2:** Layer structure of the proposed UPDT model.

Layer (Type)	Output Shape	Description
input_1 (Input Layer)	(None, 20, 7)	Input layer
lstm (LSTM)	(None, 20, 150)	LSTM encoding layer 1
lstm_1 (LSTM)	(None, 20, 50)	LSTM encoding layer 2
lstm_2 (LSTM)	(None, 20, 7)	LSTM encoding layer 3
lstm_3 (LSTM)	(None, 20, 50)	LSTM decoding layer 1
lstm_4 (LSTM)	(None, 20, 150)	LSTM decoding layer 2
lstm_5 (LSTM)	(None, 50)	LSTM decoding layer 3
dense (Dense)	(None, 6)	Output layer

**Table 3:** Dataset for model training and test.

Dataset	Normal/Fault	Number of Flights	Data Size
Train	Normal	300	X: Flight-length × 20 × 7 Y: Flight-length × 20 × 3
Test	Normal	26	X: Flight-length × 20 × 7 Y: Flight-length × 20 × 3
	Fault	3 Known Abnormal Data	X: Flight-length × 20 × 7 Y: Flight-length × 20 × 3
		120 12 Simulated Fault Modes	X: Flight-length × 20 × 7 Y: Flight-length × 20 × 3

**Table 4:** Theoretical parameter shifts at cruise conditions.

Fault Mode	EGT (°C)	FF (%)	N2 (%)
VSV closed 2°	4	0.3	1
VSV open 2°	−1	0	−1.2
VBV open 10°	11	1	0.1
1% 9th Bleed leakage	10	1.3	0.2
1% 5th Bleed leakage	9	0.8	0.2
1% 9th Bleed Cooling	6	0.5	−0.8
1% 5th Bleed Cooling	2	0.2	−0.4
−1% Fan efficiency	6	1.3	0.7
−1% Booster efficiency	4	0.4	0.1
−1% HPC efficiency	6	0.6	−0.8
−1% HPT efficiency	8	0.8	−1
−1% LPT efficiency	8	1.7	0.8

## Data Availability

Not applicable.
